# Orthodontic Bonding: Review of the Literature

**DOI:** 10.1155/2020/8874909

**Published:** 2020-07-14

**Authors:** Ali H. Alzainal, Ahmed Shehab Majud, Abdulfatah M. Al-Ani, Adil O. Mageet

**Affiliations:** Faculty of Dentistry, Ajman University, Ajman, UAE

## Abstract

**Background:**

Patients seeking orthodontic treatment are increasing, and clinicians often have to place brackets on various surfaces aside from enamel. It is crucial to know what materials or instruments are required to bond brackets to each surface.

**Objective:**

This study aims to serve as a clinical guideline for the safest and most effective approaches taken to condition various surfaces for bonding to orthodontic brackets and provide background knowledge on the subject.

**Materials and Methods:**

PubMed and EBSCO databases were searched, along with the use of Google Scholar search engine, to obtain relevant articles published in English in peer-reviewed journals, from 1955 to 2020. Keywords used were Shear bond strength; Orthodontic bracket; Base design; Etching; Sandblasting; Laser; Conditioning; Enamel; Ceramic; Porcelain; Gold; Amalgam; Composite.

**Conclusion:**

Even though orthophosphoric acid is the most widely used enamel conditioning agent, laser etching should be considered to avoid enamel decalcification. Hydrofluoric acid is the current standard for ceramic conditioning; however, its use intraorally should be minimized due to its toxicity. Orthophosphoric acid, CoJet-Sand air abrasion, and laser etching are viable alternatives for conditioning ceramic. Monobond Etch & Prime is toxic and should not be used intraorally. Composite can be conditioned by bur roughening, and the use of ceramic brackets is recommended. Amalgam and gold surfaces can be conditioned adequately by air abrasion. Despite the claims of many authors, the maximum shear forces that orthodontic brackets are subjected to are not 6–8 mega pascal (MPa). Further investigation is required in that regard. More in vivo studies need to be performed to confirm the in vitro results.

## 1. Introduction

Aesthetics have become increasingly crucial when it comes to determining the success of dental treatment, and in recent years, the demand for a better look has grown exponentially. The number of adults seeking orthodontic care increased from 14% to 27% between 2010 and 2014, based on a survey conducted by the American Association of Orthodontics back in 2015 [1], meaning that the number of orthodontic adult patients has almost doubled in 4 years and likely to continue growing as time passes.

An ideal outcome of bracket bonding to any surface should result in an attachment that is strong enough to endure the forces of orthodontic treatment and mastication without dislodgement, while at the same time be safe enough to avoid damage to the surface during debonding following the end of the treatment [2]. The desired tensile bond strength of metal brackets to tooth structure required to carry out orthodontic treatment is said to be approximately 6 MPa–8 MPa [3]. Therefore, the bond strength of brackets to the surface should not exceed the upper limit. This can be a challenging task if the bracket is to be placed on the surface of restorative material.

Although bands can be placed on restored teeth to overcome this obstacle, particularly in the posterior region, this might not be an acceptable solution in the anterior areas because of esthetic considerations. This is especially important in the interdental area due to the heightened rate at which recession occurs [4]. The banded tooth will also be predisposed to an increased accumulation of plaque [5]. Furthermore, band placement is not possible on the fixed bridge units [6].

Patients are frequently given comprehensive treatment plans involving orthodontic procedures in order to achieve the best appearance, and practitioners find themselves having to place orthodontic brackets (OB) on a variety of restorations. The purpose of this study is to serve as a clinical guideline for the safest and most effective approaches taken to condition various surfaces for bonding to OB and provide background knowledge on the subject.

## 2. Materials and Methods

PubMed and EBSCO databases were searched, along with the use of Google Scholar search engine, to obtain relevant articles published in peer-reviewed journals. Only articles published in English were included and dated from 1955 to 2020. Older articles were used either as reference for the history and background information of the materials discussed in the article, due to the scarcity of recent literature on a particular subject, or to show the consistency of results between earlier and more recent test results. References of the obtained studies were also checked to minimize the possibility of missing any relevant studies. The obtained studies were assessed by two reviewers independently. Any disagreements were settled with a discussion with the third author.

The following keywords were used: Shear bond strength; Orthodontic bracket; Base design; Etching; Sandblasting; Laser; Conditioning; Enamel; Ceramic; Porcelain; Gold; Amalgam; Composite.

## 3. Literature Review

### 3.1. Bracket Design

Bracket bases come in different shapes and forms. The design of the bracket base is a factor that influences bond strength to the attached surface. These designs include, but are not limited to, beaded, large-round-pitted, irregular, and metal mesh bases. It is difficult to determine which base design is superior as certain base designs performed particularly well with certain cements, but not as well with others [7]. The plethora of new bracket base designs and bonding cements continuously introduced to the market make it an arduous task to test all reasonably possible combinations. Further investigation is needed to determine how different base designs behave with different cements.

Another factor that influences bond strength is the material the bracket is composed of. Ceramic brackets were introduced in the mid-1980s to provide a more esthetic orthodontic treatment option [8]. Due to their growing popularity, many studies have been performed on ceramic brackets, yet the golden standard remains as metal brackets [9].

In spite of that, the ceramic brackets achieve a higher bond strength than metal brackets [10, 11], but as previously discussed, this is not always seen as an advantage. A bond strength that is too high may irreversibly damage the surface it is bonded to during debonding [11]. Coupled with the fact that the poor ductility of ceramics leads to a transfer of forces towards the underlying surfaces while debonding, greater care needs to be taken when handling ceramic brackets [12, 13].

The material of the bracket is not the only factor in regards to the risk of damaging enamel during debonding. While applying tensile forces can debond the bracket more easily, they are more likely to result in cohesive failure. A cohesive failure within the cement between the tooth/restoration surface and the bracket will result in remnants of cement on the surface after debonding. Furthermore, debonding the bracket improperly may damage the enamel. This can occur in the form of cracks or fractures macroscopically or microscopically. This can be associated with complications including poor appearance, hypersensitivity, and a higher susceptibility to pulp inflammation and caries [9].

### 3.2. Surfaces and Their Treatment Methods

#### 3.2.1. Enamel

Conditioning of enamel is necessary to obtain a surface topography capable of providing retention to the cement, as well as the fact that the enamel surface, over time, loses its properties as it reacts with the various ions and particles present in saliva after exposure for long periods [14]. Along with the impurities in enamel surface created by pores getting occupied by foreign materials, these factors necessitate the conditioning of enamel before cement application.

*(1) Orthophosphoric Acid*. Orthophosphoric acid (OPhA) is the most widely used agent to condition enamel for bonding procedures. It is not limited to orthodontics in its use. First introduced to dentistry in 1955 by Buonocore, a concentration of 85% was initially used for 30 seconds for bonding of resin to enamel [14]. Manufacturers, however, have reduced these concentrations to less than half, as they found that a concentration of 30–40% is not only safer but also produces higher bond strengths [15]. Lowering the concentration to 20% was proven to lower bond strength by a statistically significant amount [16].

The current protocol for bonding brackets to enamel is to use 37% of OPhA for 15 seconds over the surface to be etched [17–19]. The acid demineralizes enamel at varying rates, creating microporosities and uneven surface topography, allowing for the strong micromechanical adhesion [20, 21]. This is because interprismatic enamel is demineralized more readily by OPhA than prismatic enamel [22].

It should be noted that decalcifying the inorganic component of enamel makes it more susceptible to dental caries. This vulnerability is further exacerbated by the increased risk of plaque buildup around the OB [23]. Another area of concern is the retention of resin tags in enamel after the removal of cement, which could cause discolouration over time [17].

*(2) Air Abrasion*. While using acids achieve the effect of microetching, air abrasion (AA), also referred to as sandblasting, causes macroetching [24]. It was tested as a relatively conservative approach that increases surface roughness without demineralizing enamel. However, according to several authors, using 50 *μ* aluminium oxide particles produces inadequate shear bond strength (SBS) between OB and enamel [20, 23, 25].

*(3) Laser*. Lasers, first introduced in 1960 by Mainman as a ruby laser [26], are commonly seen used in dental practices, thanks to their vast array of applications [2]. The most frequently intraorally used lasers are CO_2_ and Nd:YAG lasers [27]. The mechanism through which enamel is conditioned is by its melting and recrystallization after the application of laser. An irregular surface with pores is created [28, 29], which allows for the penetration of cement [23].

The primary benefit of using lasers over acids is the effect of demineralization on enamel in the case of the latter [30, 31]. Different types of lasers are able to condition enamel for orthodontic bonding, including Nd:YAG, CO_2_, Er:YAG, and ErCs:YSGG. Though many authors have concluded that it achieves adequate bond strength [23, 32–35], some have found the contrary to be true [36–38]. It is important to note that enough power must be used for the enamel to be conditioned effectively [23]. Further studies are necessary to find the reason behind the dissimilarity between results and justify its use by clinicians.

*(4) Maleic Acid*. It is worth considering maleic acid as an alternative to OPhA etching for enamel. 10% maleic acid etching for 15 seconds creates a similar surface morphology to OPhA [39], while also removing less mineral content [22]. Few studies have been performed in the past to test the ability of maleic acid to condition enamel for OB placement [25, 40]. When comparing between OPhA and maleic acid etching, results showed that they provide a similar bond strength when testing the adherence of the cement and enamel. Considering that maleic which causes significantly less demineralization than OPhA, while also providing similar bond strength, it would be of great benefit if further studies were to be performed to confirm the validity of these results and to start a trend towards more conservative etching.

#### 3.2.2. Ceramic

Biocompatibility, a natural-looking appearance, and superior biomechanical properties are the characteristics of dental ceramics that led to their popularity as indirect restorative materials in modern restorative dentistry [41, 42]. In the earlier parts of the 20^th^ century, the first crowns with either feldspar or alumina as their components were fabricated. Due to the considerable difference between the coefficients of thermal expansion of the overlying ceramic and the underlying metal alloy, leucite was later added to the components of feldspar ceramics in the 1960s [42]. Bonding OB to a ceramic surface has a higher degree of failure when compared with bonding to enamel [43], hence necessitating finding the most effective safe bonding technique. A tooth with a ceramic restoration requires a different etching protocol than a sound tooth during the bonding of an OB because ceramics are more resistant to acids [44]. According to Kato et al., the main factor that determines the bond strength between a composite cement and a ceramic surface is the type of conditioning agent rather than the type of luting agent [45]. Therefore, the use of an ineffective technique is likely to compromise the orthodontic treatment. The surface treatment of ceramic may be mechanical, chemical, or a combination of both. The binding of organic cement to inorganic ceramic is done with the use of silane coupling agents, composed of hybrid inorganic-organo-functional trialkoxysilane monomers. This step is performed after conditioning the ceramic to provide a strong chemical adhesion to the resin cement [41, 46].

Acid is typically used to condition the surface for bonding. Two important factors to consider when conditioning a porcelain surface for bonding are the concentration of the etchant used and the amount of time that the surface is subjected to it. Increasing the exposure time will not necessarily result in a higher SBS. Contrarily, there are accounts of the SBS decreasing as a result of prolonged etching [41].

*(1) Hydrofluoric Acid*. Since the implementation of glass-based ceramics and the advances of adhesive cementation in the field, hydrofluoric acid (HFA) has been used to etch prosthodontic surfaces. The protocol for preparing a ceramic surface for bracket placement is by etching with 9.6% HFA for 1 minute; the ceramic is then rinsed with water spray. HFA application is followed by the use of silane coupling agent. Practitioners must be cautious while handling HFA as it is corrosive and toxic to living tissues [47–49]. There are no reported HFA accidents in dentistry [50]. This is likely because symptoms might not be present immediately at low concentrations, or those patients and clinicians do not attribute the symptoms to HFA exposure. The greatest danger of HFA does not stem from its low pH, but rather its toxicity mostly towards soft tissue [50].

HFA requires special care when used in the oral cavity, as skin or mucosal exposure to a concentration as low as 0.1% may cause slow-healing-burns [50]. Special considerations need to be taken when disposing HFA, especially keeping its hazardous properties in mind [48–50]. Furthermore, strong etchants such as HFA can cause irreparable surface damage to ceramic, and the resulting high bond strength of the bracket to the ceramic can put the integrity of the restoration or prosthesis at risk during the process of debonding [9, 43, 51–53].

A neutralizing agent, such as CaCO_3_ or NaOCl, is recommended to be used after the application of HFA to eliminate any remaining acidity after washing off the acid. Etching with 9.6% HFA can achieve high bond strengths between ceramic and adhesive resins by reacting with the glass phase and secondary crystalline phase, while leaving the main crystalline phase intact [44, 54], creating an irregular surface with microscopic pores that allow for micromechanical retention. The higher the crystalline content, and the lower the glass content in a ceramic, the more acid resistant it will be [44]. Even though the highest SBS is obtained using 9.6% HFA, there was no statistically significant difference in bond strengths between 9.6% and 5.0% of HFA [43, 55].

*(2) Orthophosphoric Acid*. Several authors have stated in the past that OPhA is unsuitable for etching ceramic [6, 56, 57]; however, this information is outdated. Authors have been advocating for its use as an alternative to HFA as tests, since the early 2000s have shown that OPhA is very capable of etching ceramic and obtaining adequate bond strength for orthodontic use [1, 58–63]. Furthermore, OPhA causes less surface changes when compared to HFA [61, 64, 65]. As a result, the time spent polishing the ceramic to restore it to its original state before orthodontic treatment is further decreased [1].

*(3) Laser*. With the increase in popularity of lasers in dentistry and the gradual reduction of their cost, they may be used regularly in the future to overcome the acid resistance of ceramics without having to resort to highly corrosive acids. The CO_2_ laser is well suited for the treatment of ceramic surfaces because its emission wavelength is almost totally absorbed by ceramic [2]. Conchoidal fractures are thought to facilitate mechanical interlocking between resin composite and ceramic. They occur within ceramic surfaces during ablation by a focused CO_2_ laser [2]. Several studies in vitro showed SBSs that, despite being lower than those produced by the use of HFA, are at clinically acceptable levels on different types of ceramics with the use of CO_2_ [66–70] or Nd:YAG [71, 72] lasers along with silane. However, some authors found contradictory evidence [73, 74] with the use of Nd:YAG. The difference in these results is likely due to the fact that different ceramics react differently to lasers [54].

*(4) Air Abrasion*. Mechanical preparation by the AA with aluminium oxide (Al_2_O_3_) is another proposed surface treatment method. It creates a microscopically irregular surface with a higher surface area and the pores required for micromechanical retention of the cement [44, 54, 58, 75, 76]. This provides adequate retention of OB to ceramic [43, 77–80]. However, AA is capable of causing irreversible damage to ceramic [76]. It is preferable to use low pressures (1-2 bar) using powders with particles smaller than 50 *μ*m when conditioning ceramic surfaces [54]. Interestingly, some studies from the 1990s up to more recent years have indicated that AA with 50 *μ*m aluminium oxide particles produces insufficient bond strength of ceramic to composite [56, 81, 82]. This, again, is likely because ceramics react differently to conditioning techniques [77, 83]. However, a large number of studies showed that AA using 30 *μ*m aluminium oxide with silane coupling and tribochemical coating (CoJet-Sand, 3M ESPE, Seefeld, Germany) produced very strong bonds for brackets to ceramic surfaces [54, 76, 77, 79, 80, 84].

*(5) Monobond Etch & Prime*. Some authors have recommended the use of Monobond Etch & Prime (Ivoclar Vivadent, Schaan, Liechtenstein) for orthodontic purposes [85–88]. It is a one-step conditioning agent that combines ammonium polyfluoride to etch ceramic surfaces along with silane. It was found to achieve bond strengths that are lower than conventional etching with HF and silane, but still clinically acceptable [85–88]. Even though it is a perfectly feasible option for prosthodontic use, it is highly questionable whether it should be used for orthodontic bonding of brackets, since it has been contraindicated for intraoral use by the manufacturer due to its toxicity. Unlike in the case of bracket placement, it is applied extraorally to prosthesis and washed off before concluding.

*(6) Ceramic Primer*. Burs may be used in conjunction with a ceramic primer to achieve adequate bond strength to brackets [78]. The use of a bur will deglaze the ceramic and allow the primer to react directly with the ceramic surface and not the glazing. When using the ceramic primer without deglazing the ceramic, the cement will achieve weak bond strengths [78, 89]. This method has been tested with zirconia ceramic; more tests need to be conducted for other forms of ceramic.

*(7) Burs*. Diamond bur has been tested as a conditioning technique by mechanically roughening the surface and increasing the surface area. Increasing surface roughness using burs does not increase bond strength to resin by a statistically significant amount and is not recommended as a surface conditioning technique by itself [58].

*(8) Maleic Acid*. Another interesting chemical agent is maleic acid. 10% of maleic acid has shown similar bond strengths to OPhA on ceramic [62]. More studies need to be conducted to confirm these results, as there is no reason to use more aggressive acids when milder options are just as effective.

#### 3.2.3. Composite

Clinicians are challenged with having to bond orthodontic appliances to resin composite restorations or resin laminate veneers [11]. One of the proposed ways to overcome this obstacle is prolonging the exposure of the surface to OPhA to 30–60 seconds [90–93]. Some authors advocate the use of mechanical approaches such as AA [90, 94], tungsten carbide [94], or diamond burs [11, 90]. Chemical techniques suggested including acid etching with HFA [90, 95] and silane application [91, 94–97]. HF was shown to be a suboptimal choice as a conditioning agent for composite [11], especially nanofill composite [90, 95]. Similar to ceramic, different types of composites react differently to the same conditioning method [95]. Contrary to other materials, roughening composite surfaces with a bur achieves the highest bond strengths to OB [11, 94, 98], except for nanofill composite [90]. AA showed lower bond strengths than the use of a bur [11, 94]. For nanofill composite, various conditioning methods were tested, all of which produced clinically unacceptable bond strengths. It was recommended to use AA, followed by a plastic conditioner to achieve the highest bond strength [90]. The use of silane to improve bond strength with composite was found to be unnecessary [96, 97].

Significantly higher bond strengths are achieved with the use of ceramic brackets, and its use was recommended by Eslamian et al. [11]. However, it is unclear whether the increased bond strength poses a risk of damaging the composite surface while debonding.

#### 3.2.4. Amalgam

Understandably, few tests were performed on amalgam conditioning methods for orthodontic purposes. Zachrisson et al. were the first to attempt orthodontic bonding brackets to amalgam [4]. They suggested using AA with 50 *μ*m aluminium oxide along with resin adhesives. However, this produced a mean tensile bond strength that is less than half of the mean tensile bond strength of a metal bracket to etched enamel that was used as their reference. More tests were performed using the same method and showed satisfactory results [99–101]. Roughening the amalgam surface using diamond burs was also tested but showed weaker bond strengths in all studies [4, 100]. Er:YAG laser has the ability to ablate amalgam surfaces. It increases the surface area by creating crater-like scratches that are 100 *μ*m in diameter [102, 103]. In one in vitro study, laser treatment with Er, Cr:YSGG was found to be a suitable alternative and produced higher SBSs than air-abraded amalgam [104].

#### 3.2.5. Gold

Early methods of surface preparation involved the use of greenstone [105] or roughening with sandpaper and then etching with 35% OPhA for 60 seconds [106]. AA is the current method of choice for preparation of gold surfaces [107–111]. 30 *μ*m silane-coated aluminium oxide particle AA shows promising results compared to the standard 50 *μ*m aluminium oxide particles [111]. Air drying the gold surface for the 60 seconds after silane application to allow for chemical adhesion to take place and acquire a dry operating field increases SBS [110]. While roughening the surface with a diamond bur provides adequate tensile bond strength for orthodontic purposes [107], the bond strength is significantly lower than that obtained by the AA. Tin plating the gold surface has also been suggested before AA [107], but it only provides a marginal benefit in bond strength. The results for using a metal primer on the gold surface to increase SBS are conflicting. Some authors found that it yielded poor results in bonding gold alloy to orthodontic bands [108] and metal brackets [105], while one author found it to improve SBSs [111]. According to some authors [109, 110], using adhesive primer on the base of the metal bracket achieved better results. Interestingly, light curing the resin adhesive for 40 seconds, as opposed to the manufacturer's suggestion of 20 seconds, demonstrates a significantly higher SBS [109]. It is worth keeping in mind that different ratios of gold to other metals in the alloys are being used in studies. This is likely to affect the results, making comparisons difficult.

## 4. Discussion

A systematic review on bonding OB to ceramic by Grewal Bach et al. concluded that etching with 9.6% HFA followed by silane application is the best protocol [43]. This statement is unjustified; considering that in the same study, it is mentioned that 5% HFA achieves a lower SBS, but not by a statistically significant amount. Despite achieving the highest bond strength to ceramics [43, 75], it would be safer for the patient, the practitioner, the ceramic surface, and the environment [48]—assuming the HFA is not disposed of properly—to use alternative surface treatment methods that can achieve a clinically feasible bond strength.

Panah et al. have suggested the use of patient-oriented protective measures such as neutralizing agents and rubber dams [112]. It is thought that neutralizing agents utilized in conjunction with acid etching yielded a lower SBS as it is believed that a precipitate of the HFA and the neutralizing agent interferes with the micromechanical retention of the etched surface [113–115]. Sriamporn et al. have shown that using calcium hydroxide, calcium carbonate, or calcium gluconate to neutralize the acidity of HF did not affect SBS [116].

A recent study by Lyons et al. found that there is no statistical significance in SBS resulting from the use of HFA, AA, or OPhA as lithium disilicate conditioning agents with the use of Assurance Plus (Reliance Orthodontic Products; Illinois, USA) and Transbond XT (3M Unitek Orthodontics, Minnesota, USA) [1]. This indicates that perhaps the advancement of bonding agents and types of cement, it has rendered the surface conditioning methods less impactful than they were previously.

A study by Kwak et al. to evaluate the effect of different conditioning techniques on glazed zirconia yielded interesting results [78]. They found that the use of aluminium oxide AA combined with silane resulted in higher retention than HFA with silane, but not by a statistically significant amount. The validity of these results was further reinforced by Kwak et al.'s findings. It should be noted that the difference in results was not statistically significant in Kwak et al.'s study [78].

No significant correlation was found between roughness and bond strength values [78, 117]. Perhaps, the surface area is not the main mechanical factor affecting bond strength, but rather surface architecture. Asiry et al. expressed the importance of exposing the hydroxyl ions within the ceramic surface, which are necessary for chemical bonding to the silane coupling agent [86]. Perhaps, the adequacy of conditioning methods relies on the said method's ability to expose the hydroxyl ions. Asiry et al.'s assumption is based on Matinlinna and Vallittu's study on the bonding of resin to metals with silane [118]. Though it is reasonable to believe that the factors that affect bonding to metals also apply to ceramic in this context, considering that they are both inorganic materials being bonded to an organic material.

Newman [119] was cited by several authors [35, 120–122] for stating that the maximum shear load of orthodontic forces under clinical conditions is 200 psi (1.38 MPa). However, it is unclear what these values are based on. Further tests need to be performed to confirm whether these numbers still hold true after numerous advancements in orthodontics over the past 55 years.

Reynolds et al. reported that 5.88–7.84 MPa, more commonly cited as 6–8 MPa, is the maximum amount of tensile forces that brackets are subjected to [3]. Therefore, the bond strength of the brackets to the tooth should be within that range. As for the maximum SBS that OB are subjected to, there is no reliable evidence that provides a value that can be used as a reference. Interestingly, a very large number of authors [59, 73] have used the 6–8 MPa value of tensile bond strength as a reference for the adequacy of orthodontic bonding systems, despite conducting tests to measure SBS. Shear bond strength should not be confused with tensile bond strength. Interchanging the two different types of forces is akin to measuring the kinetic energy required to cook a chicken rather than heat energy. Two studies have found that a range of 4–10 MPa is the required SBS for orthodontic treatment. However, it is unclear how this range was obtained [1, 123]. Another report of the clinically accepted SBS value was reported by Su et al. by using a lower limit of 6 MPa and an upper limit of 10 MPa. These values were obtained from the results of two different in vivo studies that aimed to compare the difference between SBS values of in vivo and in vitro studies [9]. The aim of these two studies was not to determine the maximum forces brackets are subjected to during orthodontic treatment. Further investigation is required to find the maximum intraoral SBS that OB are subjected to, even though the true value may not be far off from the currently used value.

Akova et al. stated that laboratory findings should not be interpreted as clinical recommendations because many environmental factors that potentially influence the bond strength of brackets to ceramic cannot be replicated in vitro [2]. However, they can be used to indicate which products and materials seem viable enough to include a clinical study. Despite the vast number of laboratory experiments being performed on surface conditioning methods, very few studies are performed in vivo. Even though it is difficult to design a study that isolates all but a single factor that affects bond strength, many manufacturers and clinicians would not be inclined to change their practices until clinical studies are carried out to verify the in vitro results.

## 5. Conclusion

  Optimal bracket base design is difficult to determine as each base design reacts differently according to the bracket material and resin cement used.
  OPhA is the golden standard for conditioning enamel. Many studies have shown the effectiveness of lasers as an alternative to avoid demineralization; however, more studies are needed to verify their results due to the presence of contradicting literature.
  The current protocol is to use 9.6% HFA to condition ceramic; however, 5% HFA conditions ceramic just as well. In either case, HFA is toxic, and its intraoral use should be avoided altogether. Alternatives such as OPhA, CO_2_ laser, and AA with CoJet-Sand are also effective, and their use should be considered by clinicians. Monobond Etch & Prime is toxic and should not be used intraorally to condition ceramics.
  Using ceramic brackets and roughening composite surfaces with a bur achieves the best results.
  AA is the standard technique for conditioning both amalgam and gold surfaces. More recent studies showed that laser ablation gave better results for amalgam, while using AA with CoJet-Sand improves bonding for gold alloys.
  Finally, it is unclear how the value of 6–8 MPa came to be widely regarded as the maximum value of shear forces that an OB is subjected to. Therefore, it should not be used as a reference when testing SBS of OB.

## Additional Points

*Recommendation*. Further investigation needed to find the true value of the highest shear forces OB is subjected to, and more in vivo studies are needed to confirm the findings of these in vitro results.
